# Dexmedetomidine Exerted Anti-arrhythmic Effects in Rat With Ischemic Cardiomyopathy via Upregulation of Connexin 43 and Reduction of Fibrosis and Inflammation

**DOI:** 10.3389/fphys.2020.00033

**Published:** 2020-02-07

**Authors:** Shu-jie Wu, Zhong-hao Lin, Yuan-zheng Lin, Zhi-heng Rao, Jia-feng Lin, Lian-pin Wu, Lei Li

**Affiliations:** Department of Cardiology, The Second Affiliated Hospital and Yuying Children’s Hospital of Wenzhou Medical University, Wenzhou, China

**Keywords:** dexmedetomidine, ischemic cardiomyopathy, ventricular arrhythmia, inflammation, fibrosis

## Abstract

**Background:**

Persistent myocardial ischemia post-myocardial infarction can lead to fatal ventricular arrhythmias such as ventricular tachycardia and fibrillation, both of which carry high mortality rates. Dexmedetomidine (Dex) is a highly selective α2-agonist used in surgery for congenital cardiac disease because of its antiarrhythmic properties. Dex has previously been reported to prevent or terminate various arrhythmias. The purpose of the present study was to determine the anti-arrhythmic properties of Dex in the context of ischemic cardiomyopathy (ICM) after myocardial infarction.

**Methods and Results:**

We randomly allocated 48 rats with ICM, created by persistent ligation of the left anterior descending artery for 4 weeks, into six groups: Sham (*n* = 8), Sham + BML (*n* = 8), ICM (*n* = 8), ICM + BML (*n* = 8), ICM + Dex (*n* = 8), and ICM + Dex + BML (*n* = 8). Treatments started after ICM was confirmed (the day after echocardiographic measurement) and continued for 4 weeks (inject intraperitoneally, daily). Dex inhibited the generation of collagens, cytokines, and other inflammatory mediators in rats with ICM via the suppression of NF-κB activation and increased the distribution of connexin 43 (Cx43) via phosphorylation of adenosine 5′-monophosphate-activated protein kinase (AMPK). Dex reduced the occurrence of spontaneous ventricular arrhythmias (ventricular premature beat or ventricular tachycardia), decreased the inducibility quotient of ventricular arrhythmias induced by PES, and partly improved cardiac contraction. The AMPK antagonist BML-275 dihydrochloride (BML) partly weakened the cardioprotective effect of Dex.

**Conclusion:**

Dex conferred anti-arrhythmic effects in the context of ICM via upregulation of Cx43 and suppression of inflammation and fibrosis. The anti-arrhythmic and anti-inflammatory properties of Dex may be mediated by phosphorylation of AMPK and subsequent suppression of NF-κB activation.

## Introduction

Coronary artery disease is the leading cause of morbidity and mortality around the world (Hayashi et al., 2015). Ischemic cardiomyopathy (ICM), remains a difficult problem not only because of the progression of myocardial constriction but also because of the high risk of fatal arrhythmias (Huikuri et al., 2001). There are interrelated causes contributing to the occurrence of arrhythmias after myocardial infarction, and persistent ischemia-induced inflammation and fibrosis followed by myocardial structural and electrical remodeling are crucial factors in the pathogenesis of these complications (Pahor et al., 1991; De Jesus et al., 2017; Mollenhauer et al., 2017).

Gap junctions are essential clusters of transmembrane channels that connect the cytoplasmic compartments of neighboring cells, forming sites of low-resistance electrical coupling. The uncoupling of intercellular electrical connections in the myocardium participates in the creation of the pro-arrhythmic substrate in the diseased heart, especially after myocardial infarction (Severs, 2002). Structural remodeling involves alteration in the arrangement and organization of gap junctions and genes encoding gap-junctional proteins. Its mutations in the cardiovascular system have been linked to malfunction of the heart (Dasgupta et al., 1999). Abnormal distribution of Cx43 gap junctions, a conspicuous feature of the infarct border zone of the ventricle, also contributes to heart failure (Wilkins, 1998; Wu et al., 2017). Li et al. (2018) reported that pharmacological activation of AMPK increased expression levels of Cx43 and restored the loss of functional gap junctions.

Dexmedetomidine (Dex), administered intravenously during surgery, exerted cardioprotective effects such as regulating autonomic tone and steadying perioperative hemodynamics during cardiovascular surgery (Ji et al., 2013). Dex is a highly selective, shorter-acting α-2 adrenoceptor agonist often used as an adjuvant to general anesthesia for its sedative, analgesic, anxiolytic, and hypotensive properties (Khan et al., 1999; Ji et al., 2013). The potential organ-protective effects of Dex had been a focus of attention recently (Ma et al., 2004). Dex manifested anti-inflammatory, anti-oxidative, and anti-apoptotic features in heart, brain, and lung (Chi et al., 2015; Sun et al., 2017, 2018; Deng et al., 2019). The toll-like receptor 4 (TLR4)-mediated inflammatory pathway appears to be a key line of major research focus concerning Dex, because NF-κB triggered by TLR4 activation drives pro-inflammatory cytokine gene expression, resulting in aggravated tissue damage and inflammatory reactions (Arumugam et al., 2009). Interestingly, in addition to inhibiting structural damage of organs, Dex alters the electrical activation of cardiomyocytes and causes bradycardia by attenuating L-type Ca^2+^ currents (Benitah et al., 2010). As a result, Dex exerts anti-arrhythmic efforts on acquired long QT syndrome (Benitah et al., 2010).

In our previous studies, we found that limiting the process of fibrosis and restoring the loss of gap-junctional protein inhibited the structural remodeling of the injured ventricle via suppression of persistent ischemia-induced inflammatory responses in the myocardium following myocardial infarction (Wu et al., 2017; Lin et al., 2019). Attenuated electrical remodeling, accompanied by attenuated structural remodeling, reduced the risk of fatal arrhythmias induced by continued myocardial ischemia (Wu et al., 2017; Lin et al., 2019). Therefore, in this study, in addition to investigating the anti-inflammatory properties of Dex more definitively, we emphasized the potential impact of Dex on other features such as structural remodeling, fibrosis, electrical remodeling, and arrhythmogenic inducibility. Regarding the mechanism of action, we focused on AMPK-mediated anti-inflammatory and anti-arrhythmic functions.

We hypothesized that Dex would provide cardio-protective effects on ICM after myocardial infarction. The specific aims of the study were: (1) to investigate the anti-inflammatory and anti-fibrotic properties of Dex, (2) to explore the potential anti-arrhythmic action of Dex, and (3) to reveal the underlying mechanisms behind these efforts.

## Materials and Methods

### Ethics Statement

All animal experiments in this study were conducted in accordance with the Animal Ethics Committee of Wenzhou Medical University (Number: wydw2014-0058). All experiments complied with the Guide for the Care and Use of Laboratory Animals by the National Institutes of Health (NIH). Male Sprague–Dawley (SD) rats (specific pathogen-free (SPF) class, weight 300–350 g, 10–12 weeks) were purchased from SLAC Laboratory Animal Center (Shanghai, China).

### Surgical Procedures

**FIGURE 1:**
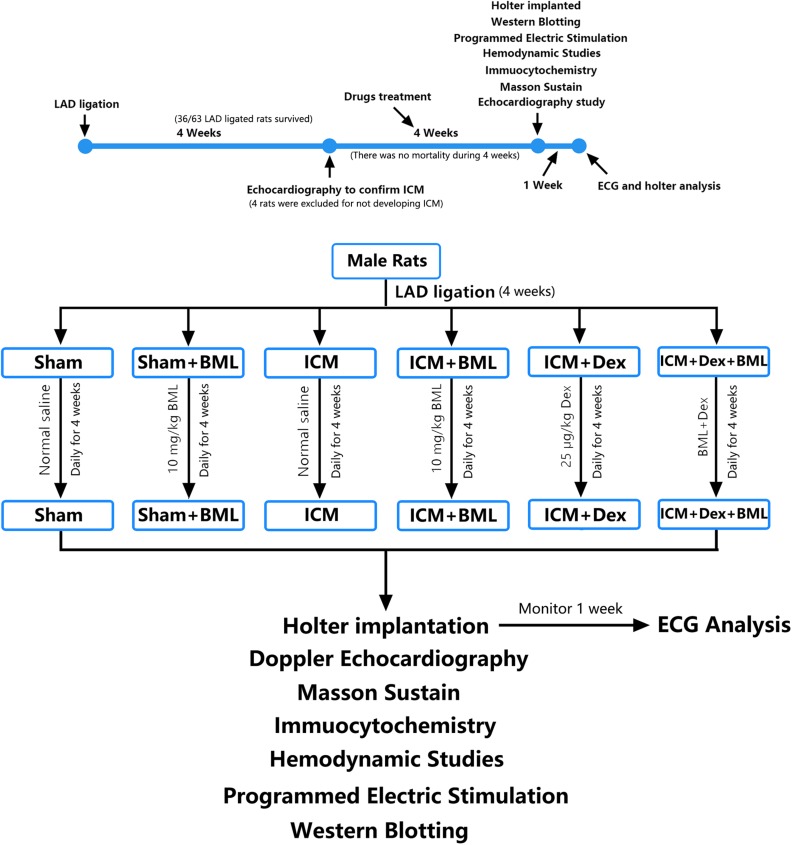
Timeline and flow chart of the complete set of experiments.

**FIGURE 2:**
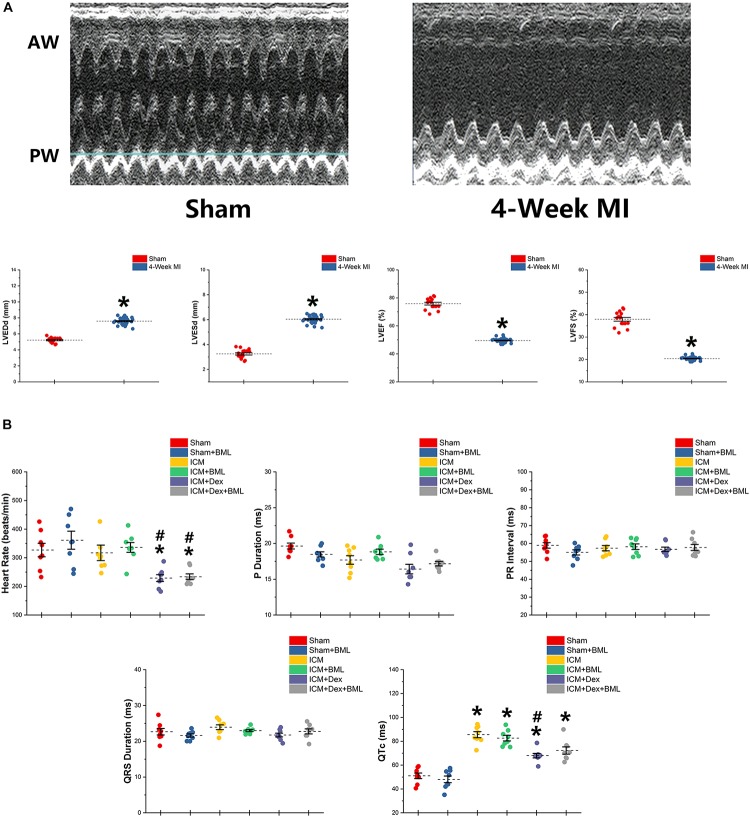
**(A)** Typical M-mode echocardiograms from sham-operated rats (Sham) and rats with 4-week ligated LAD (ICM) and changes in LVESd, LVEDd, LVEF, and LVFS between Sham group and ICM group. **(B)** ECG parameters obtained from six groups after 4-week pharmacological treatment. Thin and akinetic anterior wall (AW), hypokinetic posterior wall (PW), and dilated cardiac cavity were detected in rats with ICM. A 4-week treatment of Dex lowered the heart rate in ICM rats, while the Sham group and ICM group shared similar heat rates. ICM rats had longer QTc than did Sham rats, and Dex inhibited the effect of ischemia on the prolongation of QTc. BML had no effects on ECG parameters. Data are expressed as mean ± SD. *p < 0.05 vs. Sham group, ^#^*p* < 0.05 vs. ICM group (*n* = 8 for each group).

After anesthesia with urethane (inject intraperitoneally), rats were ventilated with a volume-controlled-mode respirator (80 strokes/min) (Inspira, Harvard Apparatus, United States). The heart was clearly exposed after left parasternotomy and the left anterior descending coronary artery was ligated 2 mm away from its origin with 6-0 Prolene suture (Wu et al., 2017). According to previous studies, the left ventricle end-diastolic dimension (LVEDd) and left ventricle end-systolic dimension (LVESd) began to increase 1 week after coronary ligation, with further progressive dilatation and reaching a plateau for up to 4 weeks (Litwin et al., 1994; Nishina et al., 2001, 2002). Thus, 4 weeks after the surgery (16/16 sham-operated rats and 36/63 LAD ligated rats survived the prior 4 weeks), surviving rats were performed with echocardiography to confirm the development of ICM (Table 1 and Figures 1, 2A). The rats that developed deteriorated cardiac contraction (LVEF around 50% or/and developing large akinetic aneurysms with some hypokinesis at non-ischemic sites) were selected (4/36 rats were excluded for not developing ICM) and divided into six groups randomly (Figure 1): (1) Sham, sham-operated rats; (2) Sham + BML, sham-operated rats with BML administration; (3) ICM, ischemic cardiomyopathy rats; (4) ICM + BML, ischemic cardiomyopathy rats with BML administration; (5) ICM + Dex, ischemic cardiomyopathy rats with Dex administration; (6) ICM + Dex + BML, ischemic cardiomyopathy rats with Dex and BML administration. The administration dosages were as follows: 10 mg/kg BML intraperitoneally (Lin et al., 2019), and 25 μg/kg Dex intraperitoneally (Cui et al., 2015; Hu et al., 2018). Rats received intraperitoneal injections of Dex or/and BML the day after echocardiography. In the ICM + Dex + BML group, BML was injected 3 h before Dex injection. All drugs were dissolved in saline containing 0.1% dimethyl sulfoxide (DMSO) and intraperitoneally injected daily. There was no mortality during the treatment phase. BML was purchased from MedChemExpress (MedChemExpress, United States), and Dex was purchased from Sigma-Aldrich (Sigma-Aldrich, China).

**TABLE 1:** Echocardiographic parameters of rats 4 weeks after surgery.

	**LVEDd (mm)**	**LVESd (mm)**	**LVEF (%)**	**LVFS (%)**
Sham	5.22 ± 0.32	3.25 ± 0.35	75.90 ± 3.76	37.94 ± 3.21
4-Week MI	7.91 ± 0.20*	6.26 ± 0.13*	50.29 ± 1.21*	20.79 ± 0.65*

*p < 0.05 vs. Sham group.

### ECG and Holter Measurements

After 4-week treatment, rats were immediately anesthetized with pentobarbital sodium (30 mg/kg, intraperitoneally). A telemetry transmitter (MicroSense, enSense, China) with two biopotential leads was implanted in the peritoneum after urethane anesthesia. Rats were housed for 1 week in cages with bottoms fitted with receivers. The digitized one-channel ECG signals were captured and analyzed by LabChart Pro ECG Analysis Module (ADInstruments, United States). Heart rate, P duration, P-R interval, and QRS duration were recorded using the PowerLab physiology system (PowerLab 8/36; AD Instruments, United States) and were also analyzed by LabChart software (AD Instruments). The QTc interval was corrected for heart rate using Framingham’s formula.

### Doppler Echocardiography

Echocardiography was performed at 4 weeks after surgery to confirm the development of ICM and was also performed after 4-week treatment (the day that telemetry transmitters were implanted). The LV end-diastolic dimension (LVEDd), LV end-systolic dimension (LVESd), left ventricular ejection fraction (LVEF), and fraction shortening (FS) were measured at the papillary muscle level, and short-axis views of M-mode tracings were recorded using Sonos 5500 system (12-MHz phased-array transducer; Philips USA, Bothell, WA, United States). All parameters were measured and recorded by an experienced technician who was blinded to study groups.

### Masson Sustain and Immunocytochemistry

Rats were sacrificed after 4-week treatment, and the LV middle ring (the middle 1/3 of the left ventricle) was isolated and embedded in paraffin as described in our previous study (Wu et al., 2017). Samples were sectioned into 5-μm-thick slices for Mallory trichrome staining. The index of fibrosis size (%) was normalized by the total area of border zone. The views of sections under the microscope were chosen randomly, and the index of fibrosis size (%) was calculated by an experienced technician who was blinded to the study groups. The index of fibrosis size was calculated using ImageJ 1.52a software (National Institutes of Health, United States).

LV middle rings (the middle 1/3 of the left ventricle) were isolated and embedded in OCT compound as described in our previous study (Lin et al., 2019). Samples were sectioned into 6-μm-thick slices. The 6-μm-thick slices were then incubated with primary antibodies overnight at 4°C after being incubated with 10% blocking serum for 30 min. FITC-conjugated secondary antibody and 4,6-diamidino-2-phenylindole dihydrochloride (DAPI, C1005 Beyotime, China) were applied for immunofluorescence. FITC and DAPI were observed with a fluorescence microscope system (DP72, Olympus, Waltham, MA, United States).

### Hemodynamic Studies

After 4-week treatment, the rats were immediately anesthetized with pentobarbital sodium (30 mg/kg, intraperitoneally). The right carotid artery was isolated, and a pressure transducer was inserted into LV via the right carotid artery. The hemodynamics data were recorded by a PowerLab physiology system and were analyzed by LabChart software.

### Programed Electric Stimulation

A vertical midline incision was made on the thorax at the end of the hemodynamic measurements. Ventricular arrhythmias were induced by programmed electrical stimulation (PES) as described previously (Wu et al., 2017). Pacing was performed by means of a programmable stimulator (PowerLab 8/36; AD Instruments). The programmed electric stimulation (PES) protocol was the same as in our previous study (Wu et al., 2017). Either no ventricular premature beats or only self-terminating salvos of <6 beats were considered “non-inducible.” Non-sustained ventricular tachyarrhythmia was defined as combining at least six consecutive ventricular premature beats and less than 15 ventricular premature beats. Sustained ventricular tachycardia consisted of more than 15 non-driven consecutive ventricular premature beats. A ventricular arrhythmias scoring system was used to evaluate the severity of ventricular tachycardia events, as described by Nguyen et al. (1998).

### Real-Time Polymerase Chain Reaction (RT-PCR)

Total RNA was extracted from the heart tissue (infarct border zone) with TRIzol Reagent (TRIzol^TM^ LS Reagent, #10296010, Invitrogen) according to the manufacturer’s protocol and was converted into cDNA with a RevertAid RT Reverse Transcription Kit (RevertAid RT Reverse Transcription Kit, K1691, Thermo Scientific). Real-time polymerase chain reaction (RT-PCR) was performed with a LightCycler 480^®^ System (LightCycler 480^®^ Instrument, Roche) by using SYBR Green I Master (LightCycler^®^480 SYBR Green I Master, #04707516001, Roche). The relative gene expressions were analyzed by the formula 2^−ΔΔ*CT*^ method. The primer sequences applied in the procedure are as follows:

Cx43: F-CTCACGTCCCACGGAGAAAA, R-CGCGATCCT TAACGCCTTTG;
TGF-β: F-CTGCTGACCCCCACTGATAC, R-AGCCCTGTA TTCCGTCTCCT;
Col-I: F-TGACTGGAAGAGCGGAGAGT, R-GATAGCGA CATCGGCAGGAT;
Col-III: F-AGTGGCCATAATGGGGAACG, R-CAGGGTTT CCATCCCTTCCG;
IL-1β: F-TAGCAGCTTTCGACAGTGAGG, R-CTCCACGG GCAAGACATAGG;
IL-6: F-CATTCTGTCTCGAGCCCACC, R-GCTGGAAG TCTCTTGCGGAG;
TNF-α: F-ATGGGCTCCCTCTCATCAGT, R-GCTTGGTGG TTTGCTACGAC;
GAPDH: F-GCAAGTTCAACGGCACAG, R-CGCCAGTAG AGACTCCACGAC.

### Western Blotting

Rats of each group were sacrificed when the 4-week treatments were finished, and hearts were snap-frozen and stored at −80°C. Myocardial tissues from the infarct border zone (2 mm away from the infarct edge) (Kohno et al., 2008) were sheared and homogenized in ice-cold lysis buffer system containing RIPA lysate (1 mL, 89900, ThermoFisher, China), protease inhibitors (10 μL, ST506-2, Beyotime, China), and phosphatase inhibitors (10 μL, P1260, Applygen, China). Subsequent steps including protein sample extraction, concentration normalization, and detection of specific protein were conducted according to our previous description (Wu et al., 2017). The details of primary antibodies are listed below: p-AMPK (Thr172; 40H9, CST, United States), AMPK (D5A2, CST, United States), Cx43 (3512, CST, United States), TGF-β (ab92486, Abcam, China), Collagen-I (14695-1-AP, Proteintech, China), Collagen-III (22734-1-AP, Proteintech, China), IL-1β (ab9722, Abcam, China), IL-6 (ab9324, Abcam, China), TNF-α (ab220210, Abcam, China), TLR-4 (ab22048, Abcam, China), MyD88 (ab2064, Abcam, China), p-P65 (Ser536; ab86299, Abcam, China), and P65 (ab16502, Abcam, China), GAPDH (ab181602, Abcam, China).

### Statistical Methods

Data are expressed as mean ± SD. All outcomes are compared among groups using one-way ANOVA followed by Dunnett’s multiple comparison test. Electrophysiological data (scoring of PES-induced arrhythmias) were compared by a Kruskal–Wallis test followed by a Mann–Whitney test. Statistical analyses were performed using SPSS 14 software (SPSS, Inc). A value of *p* < 0.05 was considered significant.

## Results

### ECG Parameters

ECG was recorded using two bio-potential leads. ECG parameters are shown in Figure 2B. There were no significant differences in P-wave duration, PR interval, and QRS duration among the six groups (*p* > 0.05). Dex lowered the heart rate in post-infarction rats, while Sham and ICM groups shared similar heat rates (Sham vs. ICM, ICM + Dex vs. ICM; Heart rate: 326.99 ± 62.22 vs. 317.28 ± 71.42, *p* > 0.05; 229.15 ± 31.53 vs. 317.28 ± 71.42, *p* < 0.05). Rats developing ICM had longer QTc intervals than did sham-operated rats, and Dex inhibited the effect of ischemia on the prolongation of QTc (Sham vs. ICM, ICM + Dex vs. ICM; QTc: 51.03 ± 6.15 vs. 85.52 ± 6.76, *p* < 0.05; 67.94 ± 4.96 vs. 85.52 ± 6.76, *p* < 0.05). BML had no effect on ECG parameters.

### Echocardiography

**FIGURE 3:**
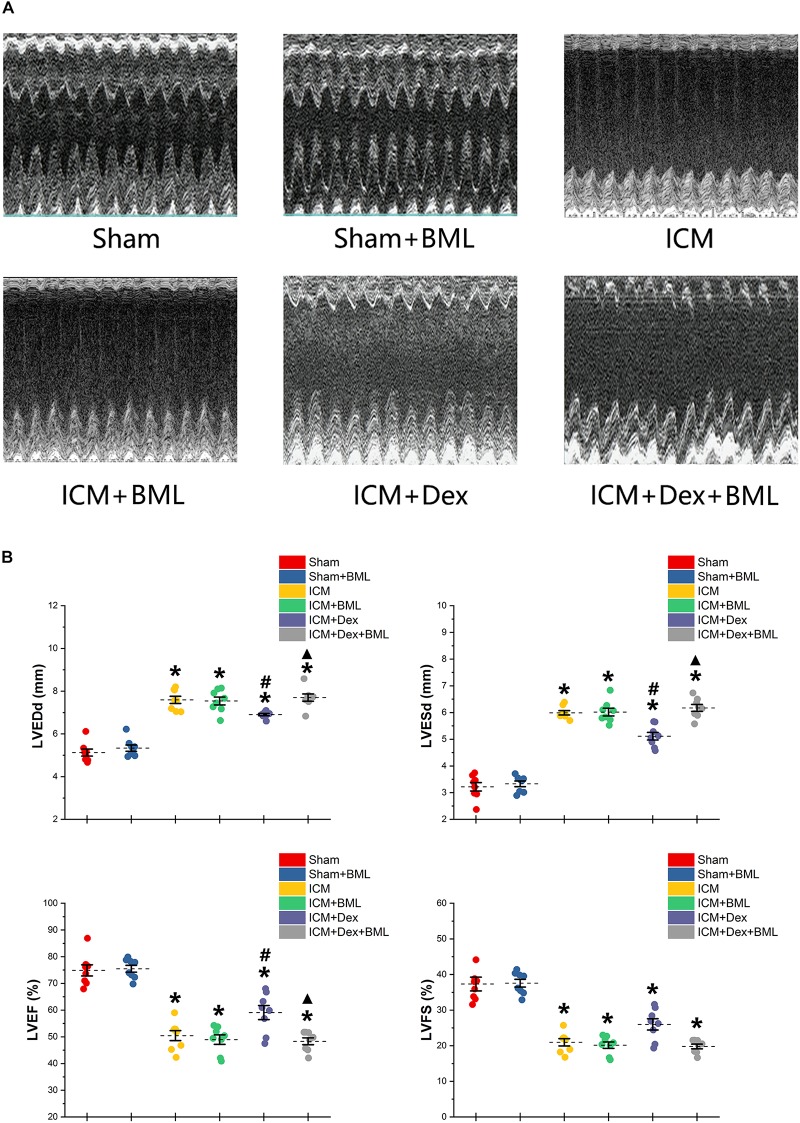
**(A)** Representative M-mode echocardiograms of short-axis views from each group after 4-week treatment. **(B)** Changes in echocardiographic properties. Larger LVEDd and LVESd and smaller LVEF and FS developed in the ICM group than in the Sham group. Lower LVEDd and LVESd and higher LVEF developed in the ICM + Dex group than in the ICM group. BML partly attenuated the effects of Dex on echocardiographic parameters. Data are expressed as mean ± SD. *p < 0.05 vs. Sham group, ^#^*p* < 0.05 vs. ICM group, ^▲^*p* < 0.05 vs. ICM + Dex group (*n* = 8 for each group).

Surviving rats developing large akinetic aneurysms accompanied by hypokinesis at non-ischemic sites with LVEF around 50% after surgery were selected and assigned for subsequent pharmacological intervention. All surviving rats underwent echocardiography again at the end of the 4-week drug administration period. Representative M-mode echocardiograms of each group are shown in Figure 3A. Larger LVEDd and LVESd and smaller LVEF and FS developed in the ICM group than in the Sham group (Table 2 and Figure 3B) (Sham vs. ICM; LVEDd, LVESd, LVEF, FS: 5.12 ± 0.43 vs. 7.59 ± 0.44, *p* < 0.05; 3.21 ± 0.42 vs. 5.99 ± 0.22, *p* < 0.05; 74.90 ± 5.49 vs. 50.45 ± 4.98, *p* < 0.05; 37.32 ± 5.12 vs. 20.96 ± 2.66; *p* < 0.05). Although Dex did not significantly change LVFS, there were lower LVEDd and LVESd and higher LVEF in the ICM + Dex group than in the ICM group (ICM + Dex vs. ICM; LVEDd, LVESd, LVEF, FS: 6.90 ± 0.14 vs. 7.59 ± 0.44, *p* < 0.05; 5.11 ± 0.38 vs. 5.99 ± 0.22, *p* < 0.05; 59.09 ± 6.98 vs. 50.45 ± 4.98, *p* < 0.05; 26.00 ± 4.16 vs. 20.96 ± 2.66; *p* > 0.05). BML partly attenuated the effects of Dex on echocardiographic parameters (ICM + Dex vs. ICM + Dex + BML; LVEDd, LVESd, LVEF, FS: 6.90 ± 0.14 vs. 7.70 ± 0.45, *p* < 0.05; 5.11 ± 0.38 vs. 6.17 ± 0.34, *p* < 0.05; 59.09 ± 6.98 vs. 48.32 ± 3.40, *p* < 0.05; 26.00 ± 4.16 vs. 19.79 ± 1.74; *p* > 0.05).

**TABLE 2:** Echocardiographic parameters of rats 4 weeks after treatment.

	**Sham**	**Sham + BML**	**ICM**	**ICM + BML**	**ICM + Dex**	**ICM + Dex + BML**
LVEDd (mm)	5.120.43	5.330.39	7.590.44*	7.540.48*	6.900.14*^#^	7.700.45*^▲^
LVESd (mm)	3.210.42	3.330.28	5.990.22*	6.010.38*	5.110.38*^#^	6.170.34*^▲^
LVEF (%)	74.905.49	75.493.42	50.454.98*	48.964.68*	59.096.98*^#^	48.323.40*^▲^
LVFS (%)	37.325.12	37.562.90	20.962.66*	20.162.40*	26.004.16*	19.791.74*
SBP (mmHg)	137.9211.34	142.7218.56	100.769.44*	108.206.44*	123.308.77^#^	104.772.23*
DBP (mmHg)	75.786.57	74.086.28	75.229.06	72.539.50	75.334.71	69.797.49
RPP (mmHg/min)	45293.7110417.46	51339.7911691.35	31900.817139.17*	36445.775845.17*	28369.815008.71*	24366.363010.88*
Pressure_Max_ (mmHg)	123.465.76	119.299.62	72.616.91*	66.897.14*	83.658.85*^#^	62.539.78*^▲^
EDP (mmHg)	6.930.72	7.319.70	15.771.62*	14.931.93*	11.541.10*^#^	16.240.73*^▲^
dP/dt_Max_ (mmHg/s)	4397.44342.63	4136.78266.42	2214.65213.53*	1995.50357.56*	2999.49335.57*^#^	2199.41324.19*^▲^
dP/dt_Min_ (mmHg/s)	−3758.86231.05	−3571.46299.28	−1437.54199.69*	−1551.43214.98*	−1994.56243.16*^#^	−1379.76317.67*^▲^

Data are expressed as mean ± SD. *p < 0.05 vs. Sham group, ^#^*p* < 0.05 vs. ICM group, ^▲^*p* < 0.05 vs. ICM + Dex group.

### Hemodynamics

**FIGURE 4:**
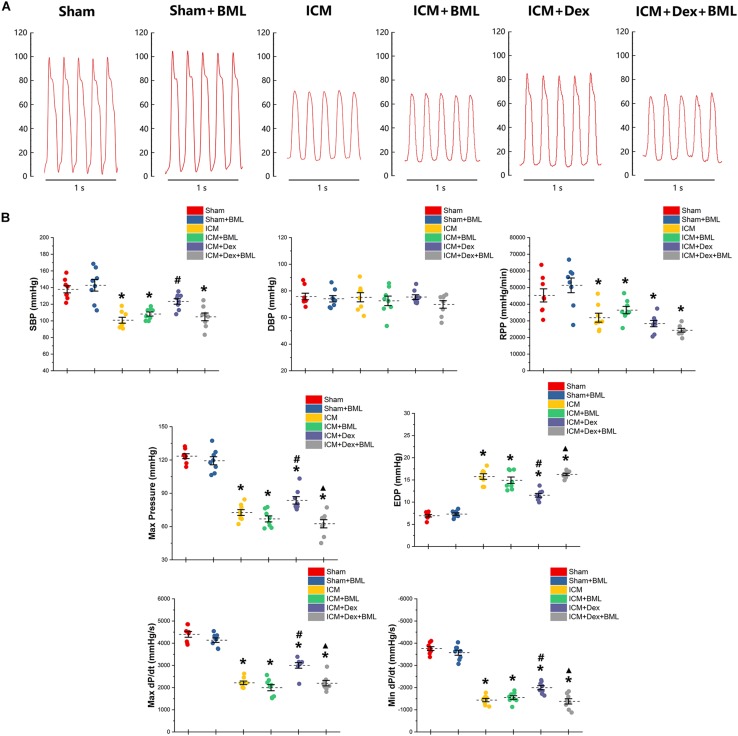
**(A)** Representative recordings of LV pressure-time curves of each group after 4-week pharmacological treatment. **(B)** Hemodynamic properties obtained from each group. Lower SBP accompanied by lower RPP was found in the ICM group than in the Sham group, while ischemic injury did not change DBP statistically. Dex restored the SBP in the ICM + Dex group. Persistent ischemia damaged cardiac contraction, manifesting as reduced Max Pressure, Max dP/dt, and Min dP/dt and increased EDP. Dex increased Max Pressure, Max dP/dt, and Min dP/dt and reduced EDP in the ICM + Dex group when compared to the ICM group. BML weakened the effects of Dex on left ventricular contraction. Data are expressed as mean ± SD. *p < 0.05 vs. Sham group, ^#^*p* < 0.05 vs. ICM group, ^▲^*p* < 0.05 vs. ICM + Dex group (*n* = 8 for each group).

Left ventricular pressure and blood pressure were measured using pressure-sensitive catheter transducers inserted into the left ventricle via the right carotid artery. The pressure waves of the left ventricle are represented in Figure 4A, and the statistical results of hemodynamic parameters are shown in Table 2 and Figure 4B. Lower SBP accompanied by lower RPP was found in the ICM group than in the Sham group, while ischemic injury did not change DBP statistically (Sham vs. ICM; SBP, DBP, RPP: 137.92 ± 11.34 vs. 100.76 ± 9.44, *p* < 0.05; 75.78 ± 6.57 vs. 75.22 ± 9.06, *p* > 0.05; 45293.71 ± 10417.46 vs. 31900.81 ± 7139.17; *p* < 0.05). Dex restored the SBP in the ICM + Dex group (ICM + Dex vs. ICM; SBP: 123.30 ± 8.76 vs. 100.76 ± 9.44, *p* < 0.05). For left ventricular pressure, persistent ischemia damaged cardiac contraction, manifesting as reduced Max Pressure, Max dP/dt, and Min dP/dt and increased EDP (Sham vs. ICM; Max Pressure, EDP, Max dP/dt, Min dP/dt: 123.46 ± 5.76 vs. 72.61 ± 6.91, *p* < 0.05; 6.93 ± 0.72 vs. 15.77 ± 1.62, *p* < 0.05; 4397.44 ± 342.63 vs. 2214.65 ± 213.53, *p* < 0.05; −3758.86 ± 231.05 vs. −1437.54 ± 199.69, *p* < 0.05). Dex increased Max Pressure, Max dP/dt, and Min dP/dt and reduced EDP in the ICM + Dex group when compared to the ICM group (ICM + Dex vs. ICM; Max Pressure, EDP, Max dP/dt, Min dP/dt: 82.65 ± 8.85 vs. 72.61 ± 6.91, *p* < 0.05; 11.54 ± 1.10 vs. 15.77 ± 1.62, *p* < 0.05; 2999.49 ± 335.57 vs. 2214.65 ± 213.53, *p* < 0.05; −1994.56 ± 243.16 vs. −1437.54 ± 199.69, *p* < 0.05). Interestingly, BML significantly weakened the effects of Dex on left ventricular pressure (ICM + Dex vs. ICM + Dex + BML; Max Pressure, EDP, Max dP/dt, Min dP/dt: 83.65 ± 8.85 vs. 62.53 ± 9.78, *p* < 0.05; 11.54 ± 1.10 vs. 16.24 ± 0.73, *p* < 0.05; 2999.49 ± 335.57 vs. 2199.41 ± 324.19, *p* < 0.05; −1994.56 ± 243.16 vs. −1379.76 ± 317.67, *p* < 0.05).

### Fibrosis on the Infarct Border Zone

**FIGURE 5:**
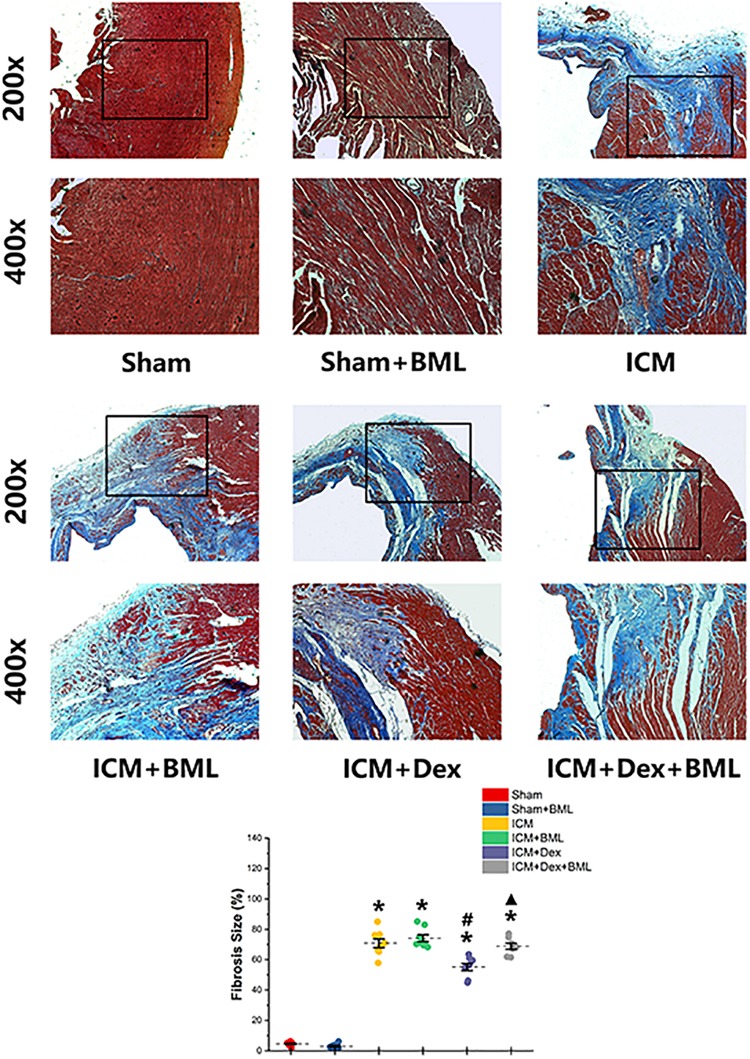
Fibrosis on infarct border zone. Significantly more fibrosis developed in the infarct border zone in ICM rats than in Sham rats. Dex treatment shrank the fibrotic area. BML attenuated the effect of Dex on fibrosis (ICM + Dex + BML vs. ICM + Dex). Data are expressed as mean ± SD. *p < 0.05 vs. Sham group, ^#^*p* < 0.05 vs. ICM group, ^▲^*p* < 0.05 vs. ICM + Dex group (*n* = 6 for each group).

The fibrosis on the infarct border zone (within 2 mm away from the infarct edge) was accessed using Mallory’s trichrome staining (Figure 5). Significantly more fibrosis developed in the infarct border zone in ICM rats than in Sham rats [Sham vs. ICM; Fibrosis size (%): 4.65 ± 1.22 vs. 70.84 ± 7.84, *p* < 0.05]. Dex treatment shrank the fibrotic area [ICM + Dex vs. ICM; Fibrosis size (%): 55.14 ± 6.19 vs. 70.83 ± 7.84, *p* < 0.05]. BML attenuated the effect of Dex on fibrosis [ICM + Dex vs. ICM + Dex + BML; Fibrosis size (%): 55.14 ± 6.19 vs. 68.80 ± 5.18, *p* < 0.05].

### Holter and PES-Induced Ventricular Tachyarrhythmia

**FIGURE 6:**
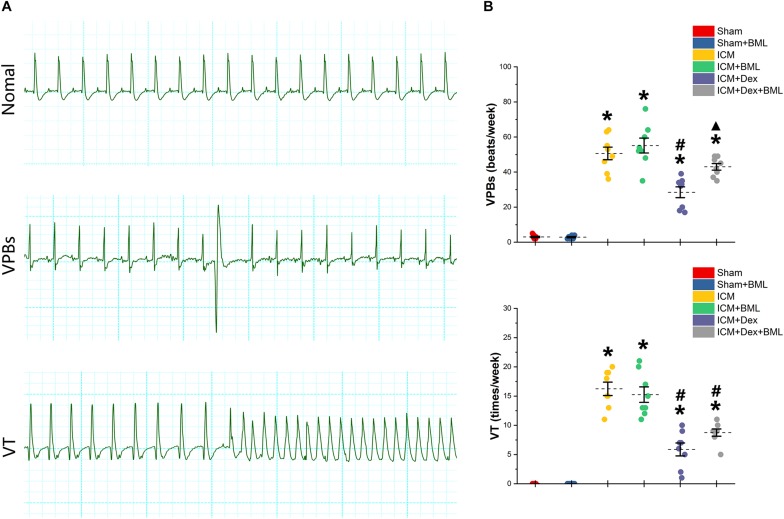
**(A)** Typical examples of ECG graphs of normal beats, ventricular premature beats (VPBs), and ventricular tachycardia (VT). **(B)** Occurrence of ventricular arrhythmias during 1 week. More VPBs and VT were found in ICM group. Dex decreased the occurrence of both VPBs and VT in the ICM + Dex group. The ICM + Dex + BML group showed more VPBs than did the ICM + Dex group. Data are expressed as mean ± SD. *p < 0.05 vs. Sham group, ^#^*p* < 0.05 vs. ICM group, ^▲^*p* < 0.05 vs. ICM + Dex group (*n* = 8 for each group).

Rats were housed in cages with bottoms fitted with receivers to capture digitized ECG signals for 1 week. Typical examples of the ECG graphs of normal beats, ventricular premature beats (VPBs), and ventricular tachycardia (VT) are shown in Figure 6A. More VPBs and VT were found in the ICM group (Sham vs. ICM; VPBs, VT: 3.00 ± 1.00 vs. 50.63 ± 9.55, *p* < 0.05; 0.00 ± 0.00 vs. 16.25 ± 3.03, *p* < 0.05). Dex administration decreased the occurrence of both VPBs and VT in the ICM + Dex group (ICM + Dex vs. ICM; VPBs, VT: 28.5 ± 8.14 vs. 50.63 ± 9.55, *p* < 0.05; 5.88 ± 2.93 vs. 16.25 ± 3.03, *p* < 0.05). The ICM + Dex + BML group showed more VPBs than did the ICM + Dex group (ICM + Dex vs. ICM + Dex + BML; VPBs: 28.5 ± 8.14 vs. 43.00 ± 5.02, *p* < 0.05) (Figure 6B).

**FIGURE 7:**
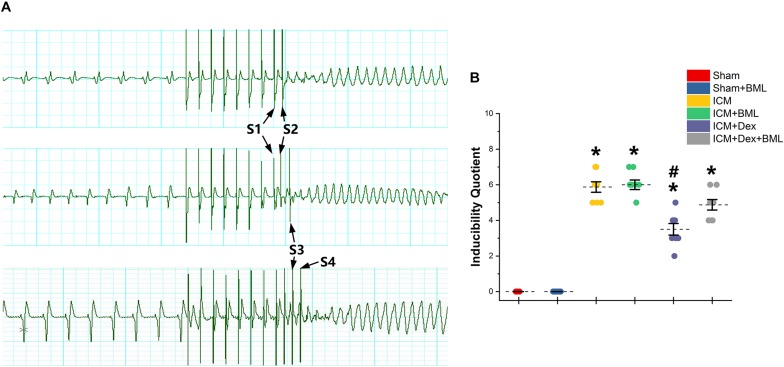
**(A)** Representative examples of sustained ventricular tachycardia induced by one (S2), two (S3), and three (S4) extra stimuli. **(B)** Inducibility quotient of ventricular arrhythmias by PES among six groups. Persistent ischemic injury increased the inducibility quotient, and Dex-treated rats had a lower quotient than did ICM rats. Data are expressed as mean ± SD. *p < 0.05 vs. Sham group, ^#^*p* < 0.05 vs. ICM group (*n* = 8 for each group).

PES-induced ventricular tachyarrhythmia is shown in Figure 7. Considering the severity of the arrhythmia induced by calculating the inducibility quotient, persistent ischemic injury increased the inducibility quotient (Sham vs. ICM; PES: 0.00 ± 0.00 vs. 5.87 ± 0.78, *p* < 0.05), and Dex-treated rats had a lower quotient than did ICM rats (ICM + Dex vs. ICM; PES: 3.50 ± 0.87 vs. 5.87 ± 0.78, *p* < 0.05).

### Distribution of Cx43

**FIGURE 8:**
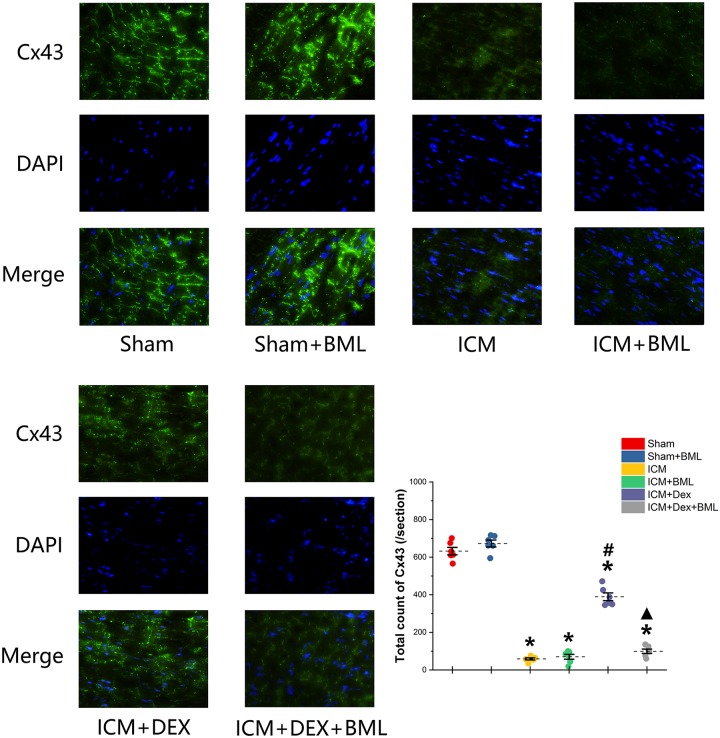
Immunofluorescence images of Cx43 from rat left ventricles (magnification x400). The ICM group had significantly less Cx43 than did the Sham group, and the distribution of Cx43 in the ICM + Dex group tended to be denser than that in the ICM group. BML attenuated this effect significantly. Data are expressed as mean ± SD. *p < 0.05 vs. Sham group, #*p* < 0.05 vs. ICM group, ▲*p* < 0.05 vs. ICM + Dex group (*n* = 6 for each group).

Cx43 located in the myocardial intercalated disk in the infarct border zone (within 2 mm away from the infarct edge) was combined with FITC-conjugated specific antibody, and FITC fluorescence was observed using a fluorescence microscope (Figure 8). The ICM group had significantly less Cx43 than did the Sham group (Sham vs. ICM; Total count of Cx43: 632.39 ± 44.53 vs. 58.86 ± 12.92, *p* < 0.05), and the distribution of Cx43 in the ICM + Dex group tended to be denser than that in the ICM group (ICM + Dex vs. ICM; Total count of Cx43: 389.25 ± 45.93 vs. 58.86 ± 12.92, *p* < 0.05). BML attenuated this effect significantly (ICM + Dex vs. ICM + Dex + BML; Total count of Cx43: 389.25 ± 45.93 vs. 99.42 ± 26.06, *p* < 0.05).

### Western Blotting and RT-PCR

**FIGURE 9:**
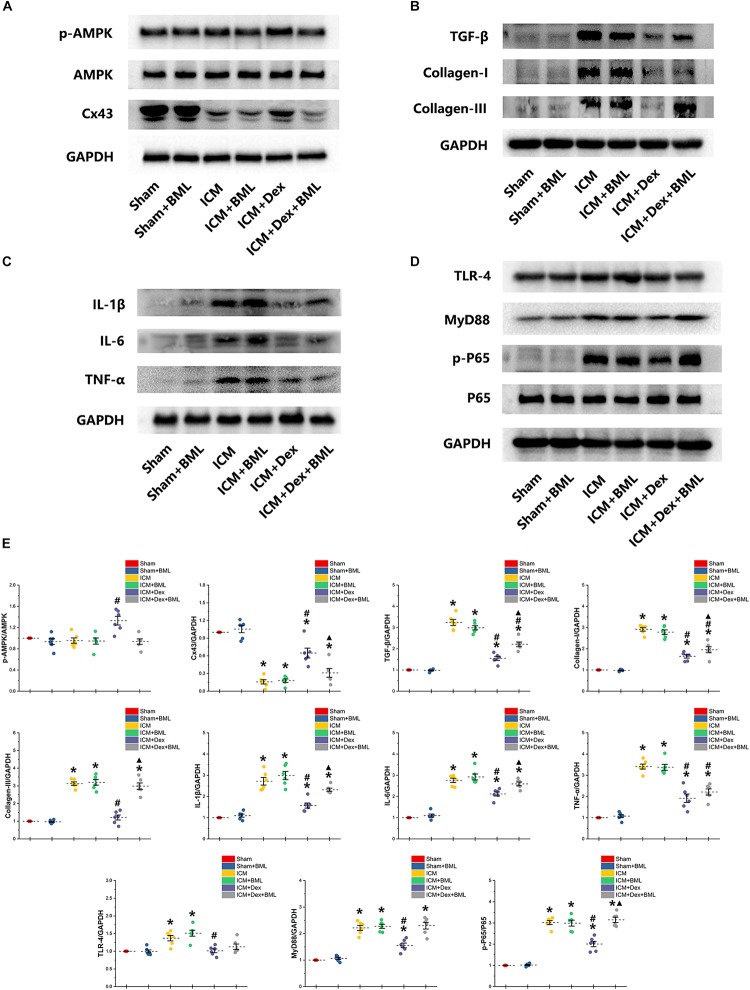
**(A–E)** Western blotting demonstrated protein expression in the AMPK pathway, Cx43, fibrosis, pro-inflammatory cytokines, and TLR-4-NF-κB pathway. Dex increased the phosphorylation of AMPK and the expression of Cx43 in the ICM + Dex group. Dex also reduced the expression of collagens, TGF-β, and pro-inflammatory cytokines. There was less activation of the TLR-4-NF-κB pathway in the ICM + Dex group than in the ICM group. BML blunted the effect of Dex on the AMPK pathway, Cx43, fibrosis, pro-inflammatory cytokines, and the TLR-4-NF-κB pathway. Data are expressed as mean ± SD. *p < 0.05 vs. Sham group, #*p* < 0.05 vs. ICM group, ▲*p* < 0.05 vs. ICM + Dex group (*n* = 6 for each group).

Western blotting demonstrated protein expression in the AMPK pathway, Cx43, fibrosis, pro-inflammatory cytokines, and TLR-4-NF-κB pathway (Figure 9). Dex significantly increased phosphorylation of AMPK and expression of Cx43 in the ICM + Dex group (ICM + Dex vs. ICM, *p* < 0.05). Dex also reduced the expression of collagens, TGF-β, and pro-inflammatory cytokines (ICM + Dex vs. ICM, *p* < 0.05). There was significantly less activation of the TLR-4-NF-κB pathway in the ICM + Dex group than in the ICM group (*p* < 0.05). BML blunted the effect of Dex on the AMPK pathway, Cx43, fibrosis, pro-inflammatory cytokines, and the TLR-4-NF-κB pathway.

**FIGURE 10:**
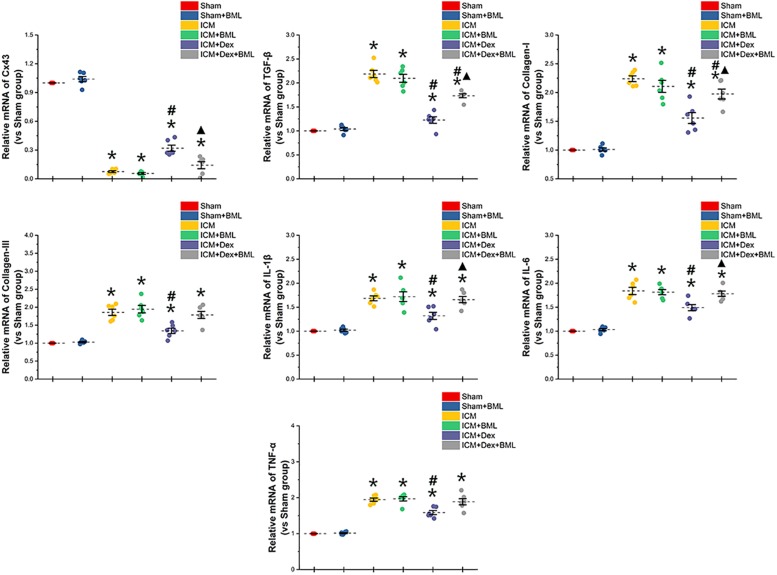
mRNA levels of Cx43-, fibrosis-, and pro-inflammatory cytokine-related proteins. Rat myocardium with ischemic injury showed increased mRNA levels of TGF-β, collagen-I, collagen-III, and pro-inflammatory cytokines and significantly lower levels of Cx43 mRNA. Rats treated with Dex showed a tendency to present lower levels of fibrosis and pro-inflammatory mRNAs than did those in the ICM group. Dex significantly increased Cx43 mRNA levels in the ICM + Dex group. BML attenuated the effects of Dex on mRNA levels. Data are expressed as mean ± SD. *p < 0.05 vs. Sham group, #*p* < 0.05 vs. ICM group, ▲*p* < 0.05 vs. ICM + Dex group (*n* = 6 for each group).

To determine whether the mechanisms of action of proteins begin at the genetic level or the post-transcriptional level, we performed RT-PCR (Figure 10). Rat myocardium with ischemic injury showed increased mRNA levels of TGF-β, collagen-I, collagen-III, and pro-inflammatory cytokines and significantly lower levels of Cx43 mRNA (Sham vs. ICM, *p* < 0.05). Rats treated with Dex showed a tendency to present lower levels of fibrotic and pro-inflammatory mRNAs than did those in the ICM group (ICM + Dex vs. ICM, *p* < 0.05). Dex significantly increased Cx43 mRNA levels in the ICM + Dex group (ICM + Dex vs. ICM, *p* < 0.05). BML attenuated the effects of Dex on mRNA levels.

## Discussion

The major findings of the present study are as follows: (1) Dex-treated rats in the context of ICM had a lower incidence of both VPBs and VT, accompanied by decreased arrhythmogenic inducibility; (2) Dex inhibited cardiac structural remodeling and improved cardiac function by suppressing fibrosis and inflammation, restoring partial loss of Cx43 during ICM; (3) Dex promoted phosphorylation of AMPK and suppression of NF-κB activation, contributing to cardio-protection.

Following myocardial infarction, inflammatory responses are required for wound healing and scar formation. Persistent and progressive inflammation and fibrosis interact coincidently with aggravating cardiomyopathy (Swirski and Nahrendorf, 2013; Wu et al., 2017; Lin et al., 2019). Activation of TLR4 on macrophages by ischemia-induced injury activates transcription factor NF-κB, which in turn leads to the upregulation and release of numerous pro-inflammatory cytokines, including IL-1β, IL-6, IFN-γ, and TNF-α, leading to exacerbation of damage to injured tissues. Inhibition of the TLR-mediated NF-κB signal pathway is among the underlying mechanisms of anti-inflammatory effects (Liu et al., 2019; Wang et al., 2019). Reducing the infiltration of neutrophils and ameliorating tissue damage by limiting excess inflammation after myocardial infarction may restore the function of the damaged heart (Liu et al., 2014; Lin et al., 2019). Regulating the polarization of macrophages after myocardial infarction also contributed to ventricular remodeling and dysfunction (Cao et al., 2019). We found that interventions involving neutrophils and inflammatory responses during ICM protected cardiac contractile function and limited histological changes (Wu et al., 2017). Based on these findings, we targeted post-myocardial infarction inflammation, a more effective approach.

Dex offers an attractive pharmacodynamic profile for procedural sedation. Based on several randomized control studies, Dex provides safe and effective sedation, facilitates extubation, and reduces delirium and myocardial injury (Ren et al., 2013; Karaman et al., 2015; Djaiani et al., 2016). In addition to these well-known properties, a recent study revealed that Dex has potential antiarrhythmic properties and can be used for the acute treatment of pediatric supraventricular tachyarrhythmias, including atrial ectopic, junctional ectopic, and reentrant type tachycardias (Chrysostomou et al., 2008). The organ-protective effects of Dex in various systems have been described in preclinical studies, and Dex exhibited promise as an anti-inflammatory agent (Hu et al., 2018; Sha et al., 2019; Yu et al., 2019). A recent study demonstrated that Dex suppressed microglial activation in ischemia-reperfusion injury of the spinal cord and suppressed inflammation by inhibiting the TLR4-mediated NF-κB signal pathway (Sun et al., 2018). In addition to inhibiting ischemia-induced inflammation in the myocardium, Dex prevented injury by activating the AMPK/PI3K/Akt/eNOS pathway (Sun et al., 2017). The aims of the present study were therefore to determine whether Dex administration would limit the inflammation occurring within ischemic myocardium and to identify the mechanisms underlying these anti-inflammatory effects with a focus on AMPK signal pathways. We found that long-term Dex treatment reduced not only the pro-inflammatory cytokine levels generated and released by ischemic injury but also the synthesis of collagens. Protein and mRNA alterations suggest that the mechanisms of changes of proteins begin at the genetic level. The hemodynamic and echocardiographic parameters, representing cardiac contraction functions that are closely relevant to myocardial fibrosis, were also improved after Dex treatment.

The results of the present study show that Dex inhibited the upregulation of the TLR4-NF-κB pathway and increased the phosphorylation of AMPK. The activation of AMPK is involved in a series of pathophysiological responses to ischemic stress; its phosphorylation suppresses regional inflammation, reduces cellular death, and inhibits cardiac structural remodeling post-myocardial infarction (Calvert et al., 2008; Peairs et al., 2009; Quan et al., 2018). The NF-κB signal pathway, which regulates inflammation and fibrosis directly, appeared be manipulated by phosphorylation of AMPK post-myocardial infarction (Zheng et al., 2018; Lin et al., 2019). A study demonstrated that AMPK suppressed the activation of NF-κB indirectly, and Sirtuin-1 (SIRT1), the Forkhead box O (F oxO) family, and peroxisome proliferator-activated receptor γ co-activator 1α (PGC-1α) might be mediators between AMPK and NF-κB (Salminen et al., 2011). To elucidate the mechanism underlying the effects of Dex treatment during ICM after myocardial infarction, we used BML, a selective AMPK antagonist. We found that BML blunted the suppression of NF-κB by Dex and subsequently weakened the anti-inflammatory and anti-fibrotic efforts of Dex on ICM. Based on these findings, we believe that the Dex-related cardioprotective effects, manifested as reduced inflammation, limited fibrosis, and improved cardiac contraction function, are related to the downregulation of NF-κB by activating AMPK.

Fatal ventricular arrhythmias, including ventricular tachycardia and fibrillation accompanied by severe heart failure, are major causes of mortality in patients after myocardial infarction (Huikuri et al., 2001; Hayashi et al., 2015). Surviving cardiomyocytes in the infarct border zone undergo dramatic electrophysiological remodeling in addition to the development of fibrotic scars, creating substrates for ventricular arrhythmia. Following myocardial infarction, compact fibrotic tissues, as well as interstitial fibrosis, develop adjacent to and outside the infarct, generating fatal arrhythmias. The former serves as an insulted area where re-entrant arrhythmias can anchor and convert to sustained ventricular tachycardia (Ripplinger et al., 2009); the latter is more arrhythmogenic, serving as a trigger (Dhein et al., 2014; Nguyen et al., 2014). Importantly, pro-inflammatory cytokine levels are elevated in the myocardium following myocardial infarction (including TNF-α, IL-1β, and IL-6), producing electrophysiological changes, suggesting that the persistent existence of inflammation is an important contributor to arrhythmia. Pro-inflammatory cytokines such as IL-1β contribute to defective excitation-contraction coupling and arrhythmogenesis in the post-myocardial infarction heart (De Jesus et al., 2017). In our study, the Dex-treated group (ICM + Dex) showed lower pro-inflammatory cytokine levels and fibrosis in the infarct border zone than in the ICM group. This finding suggests that the antiarrhythmic effect of Dex is achieved, at least in part, by its anti-inflammatory and anti-fibrotic effects. Although some studies reported that electrophysiological changes in the border zone resulted from abnormalities of ion channels, reduced expression or lateralization of Cx43 also led to triggered activity (Yao et al., 2003; Schultz et al., 2019). Interestingly, Schultz et al. (2019) recently reported that, at the infarct border zone, Cx43 reduction led to decrement in cardiac conduction velocity as well as possible pro-arrhythmogenic heterocellular coupling between cardiomyocytes and myofibroblast in the fibrotic area. Furthermore, the deficiency of gap-junctional proteins, localized in intercalated disks especially including Cx43, aggravated disrupted cardiac remodeling in post-myocardial infarction and directly increased the risk of fatal arrhythmias (Severs, 2002; Van Rijen et al., 2004; Yanni et al., 2011). In the present study, we found that Dex increased the distribution of Cx43 in the intercalated disk and reduced the incidence of both VPBs and VT accompanied by decreased arrhythmogenic inducibility; BML attenuated the effect of Dex on Cx43. Therefore, we speculate that AMPK might be critically involved in the anti-arrhythmic effect of Dex during ICM. The heart rate was significantly reduced by Dex administration, suggesting that the change in heart rate might be a confounder for the attenuation of structural remodeling and arrhythmias, because an independent relationship between pure reduction in heat rate and improvements of structural and electrical remodeling was identified (Verrier and Tan, 2009). In our study, despite the fact that BML did not change heart rate (ICM + Dex + BML vs. ICM + Dex), it weakened the protective effect of DEX on cardiac structural and electrical remodeling, suggesting that the change in heart rate might not be a major cause for remodeling and arrhythmias.

Applied to facilitate analgesia and anesthesia, Dex is not limited to its interactions with α_2_-adrenergic receptors. Dex also attenuates L-type calcium currents in rat ventricular myocytes, possibly contributing to its negative effects on the activation of cardiac electric activity and contractility of the myocardium (Zhao et al., 2013). Dex may provoke bradycardia or hypotension via modification of the human α-subunit Nav1.5 of the cardiac sodium channel. Such modification potentially contributes to both pro-arrhythmic and anti-arrhythmic effects (Jakob et al., 2012; Stoetzer et al., 2016). Interestingly, there were no differences, except for heart rate, between the Sham and Sham + Dex group (sham-operated rats with Dex administration) in terms of ECG parameters and echocardiography (Table 3). This suggests that Sham + Dex should not be used as a follow-up experimental group. We found that Dex reduced the occurrence of ventricular premature beats and ventricular tachycardia, as well as decreasing the inducibility quotient of ventricular arrhythmias by PES. BML partly attenuated the effects of Dex on arrhythmogenicity, while no alterations of ECG parameters (Heart rate, P duration, RP interval, QRS duration, and QTc) were found after BML administration. These findings suggest that Dex-induced bradycardia (lower heart rate in the ICM + Dex group) did not involve the AMPK signal pathway. Tsutsui et al. (2012) reported that Dex prolonged the QTc interval in a rabbit model. In the present study, we found that Dex shortened the prolonged QTc accompanied by a lower heart rate. We could not determine the underlying mechanism of these varying results; nevertheless, Dex may have improved structural and electrical remodeling in ICM rats. Interestingly, our findings of contraction of the myocardium in ICM being restored after Dex treatment were inconsistent with the results of a previous study (Jakob et al., 2012).

**TABLE 3:** Echocardiographic and ECG parameters of Sham and Sham + Dex group.

	**Sham**	**Sham + Dex**
LVEDd (mm)	5.120.43	5.330.39
LVESd (mm)	3.210.42	3.330.28
LVEF (%)	74.905.49	75.493.42
LVFS (%)	37.325.12	37.562.90
Heart Rate (beats/min)	326.9962.22	233.0919.56*
P Duration (mm)	19.641.10	20.401.78
PR Interval (mm)	58.944.15	55.995.11
QRS Duration (mm)	22.662.38	19.983.77
QTc (mm)	51.036.15	45.513.06

Data are expressed as mean ± SD. *p < 0.05 vs. Sham group.

Taken together, our findings suggest that Dex provides cardioprotective effects on ICM after myocardial infarction. Dex confers anti-arrhythmic effects via the upregulation of Cx43 and the suppression of fibrosis and inflammation. Dex promoted phosphorylation of AMPK and suppressed NF-κB activation, resulting in improved cardiac remodeling either structurally or electrically. Our observations may help in the development of novel therapeutic strategies for the arrhythmia in patients with ICM. Our results also provide a basis for testing the safety of anesthetic drug selection during post-myocardial infarction.

### Limitations

Although rodents are widely used in research cardiac arrhythmia, rodents are not ideal animal models for research in this field, given the differences in heart size, heart rate, and action potential configuration when compared to humans.

## Data Availability Statement

All datasets generated for this study are included in the article/supplementary material.

## Ethics Statement

The animal study was reviewed and approved by the Animal Ethics Committee of Wenzhou Medical University (Number: wydw2014-0058).

## Author Contributions

All authors listed have made a substantial, direct and intellectual contribution to the work, and approved it for publication.

## Conflict of Interest

The authors declare that the research was conducted in the absence of any commercial or financial relationships that could be construed as a potential conflict of interest.
